# Reversibility of *β*-Cell-Specific Transcript Factors Expression by Long-Term Caloric Restriction in db/db Mouse

**DOI:** 10.1155/2016/6035046

**Published:** 2016-02-21

**Authors:** Chunjun Sheng, Feng Li, Ziwei Lin, Manna Zhang, Peng Yang, Le Bu, Hui Sheng, Hong Li, Shen Qu

**Affiliations:** Department of Endocrinology and Metabolism, Shanghai Tenth People's Hospital, Tongji University, 301 Middle Yanchang Road, Shanghai 200072, China

## Abstract

Type 2 diabetes (T2D) is characterized by *β*-cell dedifferentiation, but underlying mechanisms remain unclear. The purpose of the current study was to explore the mechanisms of *β*-cell dedifferentiation with and without long-term control of calorie intake. We used a diabetes mouse model (db/db) to analyze the changes in the expression levels of *β*-cell-specific transcription factors (TFs) and functional factors with long-term caloric restriction (CR). Our results showed that chronic euglycemia was maintained in the db/db mice with long-term CR intervention, and *β*-cell dedifferentiation was significantly reduced. The expression of Glut2, Pdx1, and Nkx6.1 was reversed, while MafA expression was significantly increased with long-term CR. GLP-1 pathway was reactivated with long-term CR. Our work showed that the course of *β*-cell dedifferentiation can intervene by long-term control of calorie intake. Key *β*-cell-specific TFs and functional factors play important roles in maintaining *β*-cell differentiation. Targeting these factors could optimize T2D therapies.

## 1. Introduction

Type 2 diabetes (T2D) is characterized by *β*-cell dysfunction. In addition to cell-autonomous defects that can be demonstrated long before disease onset [1], there are cell-nonautonomous contributors to *β*-cell dysfunction, such as disturbance in *α*- and *β*-cell interactions [2], pancreatic ectopic lipid deposition [3, 4], and islet fibrosis [5]. Targeting these cell-nonautonomous defects might optimize diabetes therapies.

Increasing evidence has identified transcription factors critical for the maintenance of a mature *β*-cell phenotype. The inactivation of specific *β*-cell transcription factors results in the dedifferentiation of *β*-cells [6], although the molecular mechanisms remain unknown. Strikingly, additional experiments have shown that *β*-cell dedifferentiation is a reversible and dynamic state and that intensive insulin therapy leads to redifferentiation to mature *β*-cells [7]. Thus, the identification of interventions that could reduce *β*-cell dedifferentiation or help dedifferentiated *β*-cell revert to functional *β*-cells deserves further study.

In db/db mice, a classical obese diabetes model, significant *β*-cell dedifferentiation is observed at 3 months of age [8]. We used this model to study *β*-cell dedifferentiation and redifferentiation. Our results show that although *β*-cell dedifferentiation started, after long-term caloric restriction (CR) intervention, *β*-cell function is significantly ameliorated and *β*-cell dedifferentiation is significantly reduced. Intriguingly, the expression of Glut2 and *β*-cell-specific transcription factors (TFs) Pdx1 and Nkx6.1 can be fully reversed to normal levels. Moreover, GLP-1 pathway was also reactivated after long-term CR. In the present study, we examined the nature of *β*-cell dedifferentiation in the natural history of T2D with and without caloric restriction intervention, shedding light on the pathogenesis of T2D and how mature *β*-cells differentiate and maintain their functions.

## 2. Materials and Methods

### 2.1. Animals

The C57BLKS/J-Leprdb/Leprdb (db/db) and C57BLKS/J-Leprdb/m (db/m) male mice were purchased from the Shanghai Laboratory Animal Center, Chinese Academy of Sciences (SLAC, CAS). All of the mice were housed in pathogen-free facilities with a 12 h light/dark cycle. Male 12-week-old db/db mice were randomly assigned to an* ad libitum* diet with free access to regular chow (db/db-F), or db/db-R, receiving limited food supply (0.1 g/g body weight/day) for 3 months. Food was added for db/db-R mice at fixed time every day (12 o'clock). As control, male db/m mice were also given free access to regular chow (db/m-F) for 3 months. All of the animal experiments were conducted in accordance with the* Guide for the Care and Use of Laboratory Animals* published by the National Institutes of Health. Animal use has been reviewed and approved by the Animal Ethical and Welfare Committee (AEWC) of Tongji University.

### 2.2. Glucose Tolerance Test and Metabolic Measurements for Mice Experiments

The mice were fasted for 12 h before the glucose tolerance test. The mice were injected intraperitoneally with 1 g kg^−1^ glucose. The glucose measurements were taken up to 2 h after injection using OneTouch Ultra glucometers (LifeScan). The serum insulin levels were measured by a mouse insulin ELISA kit (Crystal Chem).

### 2.3. Histologic and Immunostaining Analyses

Pancreata were harvested and fixed in 4% buffered formaldehyde. The immunohistologic analyses were performed on paraffin serial sections, as described previously [9]. The antibodies used for the immunochemistry and immunofluorescence assays are the following: polyclonal rabbit anti-Pdx1 antibody (1 : 3000, Abcam), polyclonal rabbit anti-MafA antibody (1 : 2000, Abcam), polyclonal rabbit anti-Nkx6.1 antibody (1 : 200, Novus Biologicals), polyclonal rabbit anti-Glut2 antibody (1 : 400, Abcam), monoclonal rabbit anti-PKC*ζ* antibody (1 : 100, Abcam), monoclonal mouse anti-insulin antibody (1 : 1000, Sigma), polyclonal rabbit anti-glucagon antibody (1 : 200, Cell Signaling Technology), polyclonal rabbit anti-ChrA antibody (1 : 200, Abcam), and polyclonal rabbit anti-Foxo1 antibody (1 : 100, Cell Signaling Technology). The secondary antibodies used in the immunofluorescence staining assays were purchased from Invitrogen. The images were acquired using a Zeiss confocal microscope or an Olympus system.

### 2.4. Isolation of Mouse Pancreatic Islets and Glucose-Stimulated Insulin Secretion (GSIS)

Pancreatic islets were isolated from mice at 2–15 months of age as previously described [9]. Briefly, the pancreases were digested with collagenase and dissociated vigorously by mechanical pipetting. The islets were “hand-picked” from dark-field dishes under a dissecting microscope and pooled for further analysis.

Islets were incubated over a period of 60 min in 1 mL Krebs-Ringer bicarbonate Hepes buffer (KRBH, 140 mM NaCl, 3.6 mM KCl, 0.5 mM NaH_2_PO_4_, 0.5 mM MgSO_4_, 1.5 mM CaCl_2_, 2 mM NaHCO_3_, 10 mM Hepes (pH 7.4), and 0.25% BSA) containing 2.8 mM/L glucose or 16.7 mM/L glucose. Experiments were conducted with three to five tubes for each condition. The insulin levels in the supernatant were measured by a mouse insulin ELISA kit (Crystal Chem).

### 2.5. Quantitative PCR Analysis

The total RNA extraction was performed on hand-picked islets using an RNeasy kit (Qiagen) according to the manufacturer's instructions. Quantitative real-time polymerase chain reactions (PCRs) were performed as previously described [9]. The following primer pairs were used in this study:

 MafA-fw: 5′-AGGAGGAGGTCATCCGACTG-3′.
 MafA-rev: 5′-CTTCTCGCTCTCCAGAATGTG-3′.
 Nkx6.1-fw: 5′-CTGCACAGTATGGCCGAGATG-3′.
 Nkx6.1-rev: 5′-CCGGGTTATGTGAGCCCAA-3′.
 Pdx1-fw: 5′-CCCCAGTTTACAAGCTCGCT-3′.
 Pdx1-rev: 5′-CTCGGTTCCATTCGGGAAAGG-3′.
 GLP-1(Gcg)-fw: 5′-TTACTTTGTGGCTGGATTGCTT-3′.
 GLP-1(Gcg)-rev: 5′-AGTGGCGTTTGTCTTCATTCA-3′.
 GLP-1R-fw: 5′-ACGGTGTCCCTCTCAGAGAC-3′.
 GLP-1R-rev: 5′- ATCAAAGGTCCGGTTGCAGAA-3′.
 Glut2-fw: 5′-TCAGAAGACAAGATCACCGGA-3′.
 Glut2-rev: 5′-GCTGGTGTGACTGTAAGTGGG-3′.
 PKC*ζ*-fw: 5′-GCGTGGATGCCATGACAAC-3′.
 PKC*ζ*-rev: 5′-AATGATGAGCACTTCGTCCCT-3′.

### 2.6. Statistical Analysis

All of the results are reported as the means ± standard errors of the mean. Differences for continuous variables were assessed by performing *t*-test, ANOVA, or ANCOVA as appropriate. Bonferroni correction was used for the post hoc analyses; *P* values less than 0.05 were considered significant. All of the analyses were performed using the GraphPad Prism software (GraphPad Software Inc.).

## 3. Results

### 3.1. *β*-Cell Function Is Significantly Ameliorated following Long-Term Calorie Restriction (CR)

**Figure 1:**
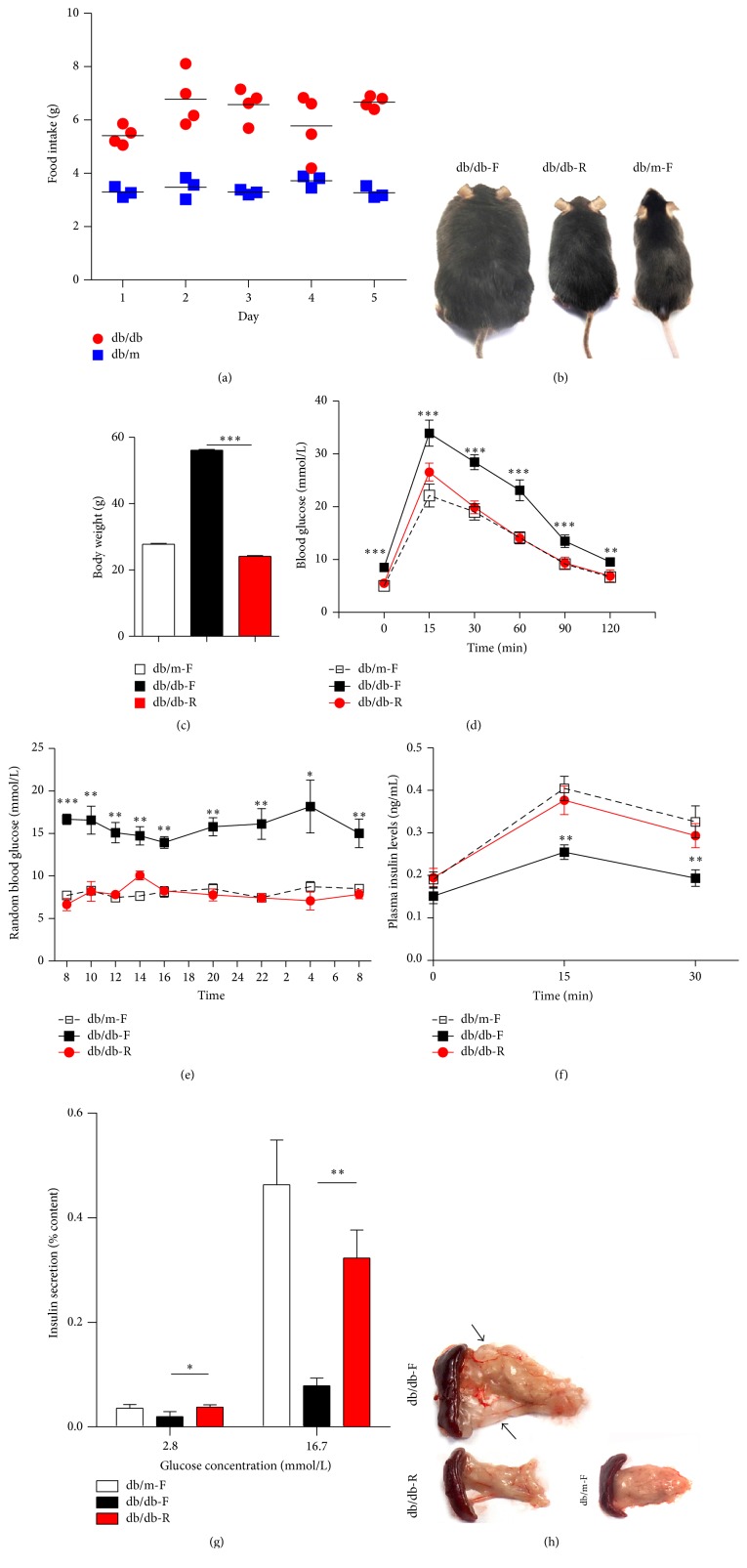
Improvement of *β*-cell function after long-term caloric restriction (CR). (a) Continuous food intake monitoring showed that increased food intake was observed in db/db mice compared with db/m mice at 3 months of age. (b-c) db/db-R mice were healthy and their weights were significantly low compared with db/db-F mice. (d) Intraperitoneal glucose tolerance test (IPGTT) was performed and blood glucose was measured at 0, 15, 30, 60, 90, and 120 min in the db/m-F, db/db-F, and db/db-R mice. (e) Twenty-four-hour dynamic blood glucose monitoring was performed on db/m-F, db/db-F, and db/db-R mice. (f) Plasma insulin levels were measured at 0, 15, and 30 min after IPGTT in db/m-F, db/db-F, and db/db-R mice. (g) Insulin secretion from islet cells isolated from db/m-F, db/db-F, and db/db-R mice was measured after the glucose-stimulated insulin secretion (GSIS) test. (h) The adipose tissue around the pancreas was examined in db/m-F, db/db-F, and db/db-R mice. The* arrows* indicate the adipose tissue. ^*∗*^ *P* < 0.05, ^*∗∗*^ *P* < 0.01, and ^*∗∗∗*^ *P* < 0.001. The data shown represent three independent experiments. db/db-F and db/db mice given free access to regular chow; db/db-R and db/db mice receiving restricted food supply; db/m-F and db/m mice given free access to regular chow.

Before CR, increased food intake was observed in db/db mice compared with db/m mice (Figure 1(a)). After 3 months of CR, db/db-R mice are healthy and their body weights are significantly reduced compared with db/db-F mice (Figures 1(b)-1(c)). An intraperitoneal glucose tolerance test (IPGTT) showed that db/db-R mice, compared with db/db-F mice, exhibited a much improved IPGTT curve, although 15 min after glucose loading a significant increase in blood glucose levels was still observed in the db/db-R mice compared with the db/m-F mice (Figure 1(d)). Additionally, dynamic glucose monitoring showed that nearly normal random blood glucose was observed in the db/db-R mice compared with the db/db-F mice (Figure 1(e)). IPGTT revealed a significant increase in plasma insulin levels in db/db-R mice compared with db/db-F mice (Figure 1(f)). A glucose-stimulated insulin secretion (GSIS) test showed that insulin secretion from isolated islets in db/db-R mice was significantly increased at both low and high glucose concentrations compared with db/db-F mice (Figure 1(g)). Adipose tissue deposited around the pancreas was significantly reduced in the db/db-R mice compared with the db/db-F mice (Figure 1(h)).

### 3.2. Long-Term CR Results in the Normal *α*-Cell Quantities and Arrangements

**Figure 2:**
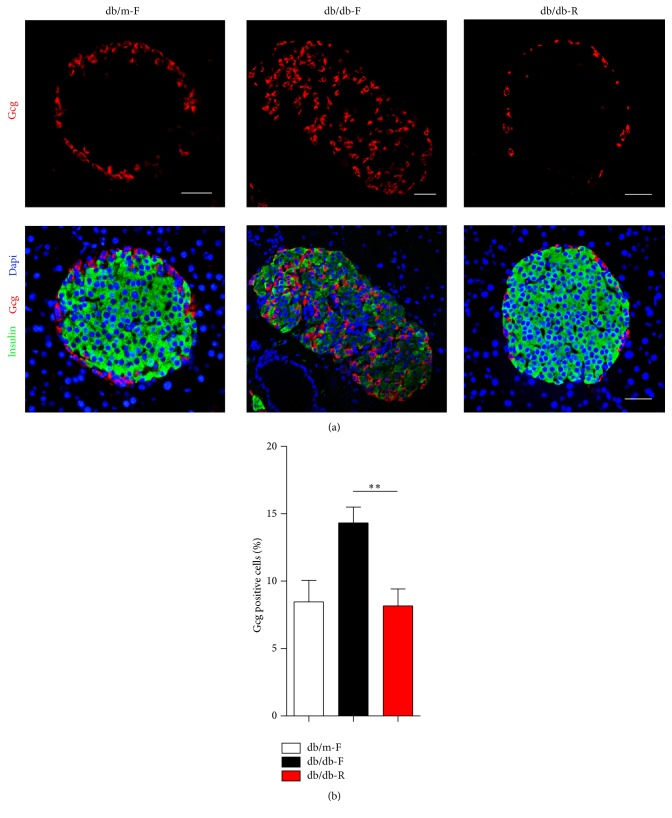
Islet morphology in db/m-F, db/db-F, and db/db-R mice. (a) Representative islet cells from db/m-F, db/db-F, and db/db-R mice stained for insulin (red) and glucagon (Gcg, green). Increased intraislet *α*-cells were observed in db/db-F mice, while most of the *α*-cells located in the mantle of islet cells were observed in db/m-F and db/db-R mice. (b) Proportion of Gcg positive cells observed in the islet cells in db/m-F, db/db-F, and db/db-R mice. ^*∗∗*^ *P* < 0.01. db/db-F and db/db mice given free access to regular chow; db/db-R and db/db mice receiving restricted food supply; db/m-F and db/m mice given free access to regular chow. Scale bars: 25 *μ*m.

To examine the effects of CR on *β*-cells, pancreatic sections were analyzed by immunohistochemistry. It was observed that insulin immunoreactivity in the *β*-cells was reduced in combination with a relative increase in the number of intraislet *α*-cells in db/db-F mice. In contrast, in db/db-R mice, insulin immunoreactivity was significantly increased and intraislet *α*-cells were significantly reduced compared with db/db-F mice (Figures 2(a)-2(b)). The quantity and arrangement of *α*-cells were normal in the db/db mice at 12 weeks of age (data not shown), suggesting that long-term CR could prevent the change in islet morphology during T2D progression.

### 3.3. *β*-Cell Dedifferentiation Is Reduced after Long-Term CR

**Figure 3:**
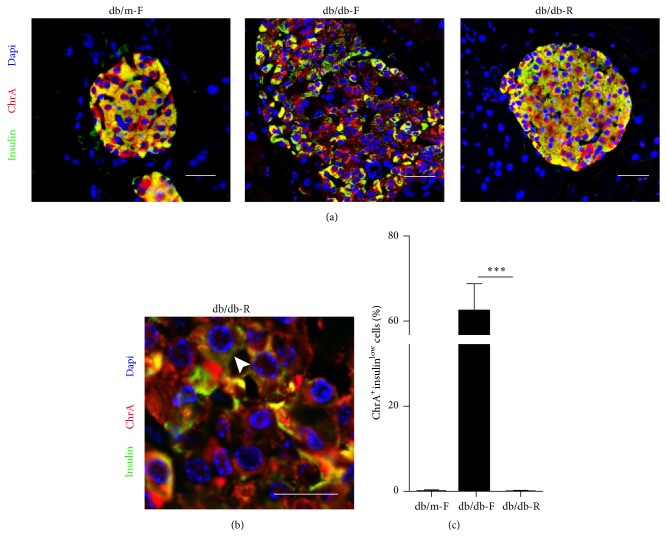
*β*-cell dedifferentiation in db/m-F, db/db-F, and db/db-R mice. (a) Immunofluorescence analysis of Chromogranin A (ChrA) and insulin expression in db/m-F, db/db-F, and db/db-R mice. (b) ChrA-positive cells with low levels of insulin expression (ChrA^+^insulin^low^) were significantly increased in the db/db-F mice. The* arrow* indicates the ChrA^+^insulin^low^ cell. (c) Proportion of ChrA^+^insulin^low^ cells observed in the islet cells in db/m-F, db/db-F, and db/db-R mice. ^*∗∗∗*^ *P* < 0.001. db/db-F and db/db mice given free access to regular chow; db/db-R and db/db mice receiving restricted food supply; db/m-F and db/m mice given free access to regular chow. Scale bars: 25 *μ*m.

Chromogranin A (ChrA) is a committed endocrine cell marker [10]. Immunofluorescence staining showed that increased ChrA^+^insulin^−^ cells were observed in db/db-F mice (Figure 3(a)), suggesting their endocrine destiny. As expected, ChrA-positive cells with low levels of insulin expression (ChrA^+^insulin^low^) were significantly increased in db/db-F mice, while this could barely be observed in db/db-R mice (Figures 3(b)-3(c)). The ChrA^+^insulin^low^ cells had lost insulin expression and were undergoing dedifferentiation. Therefore, *β*-cell dedifferentiation could be prevented by long-term CR.

### 3.4. The Expression of Glut2 and Specific *β*-Cell Transcription Factors Is Reversed after Long-Term CR

**Figure 4:**
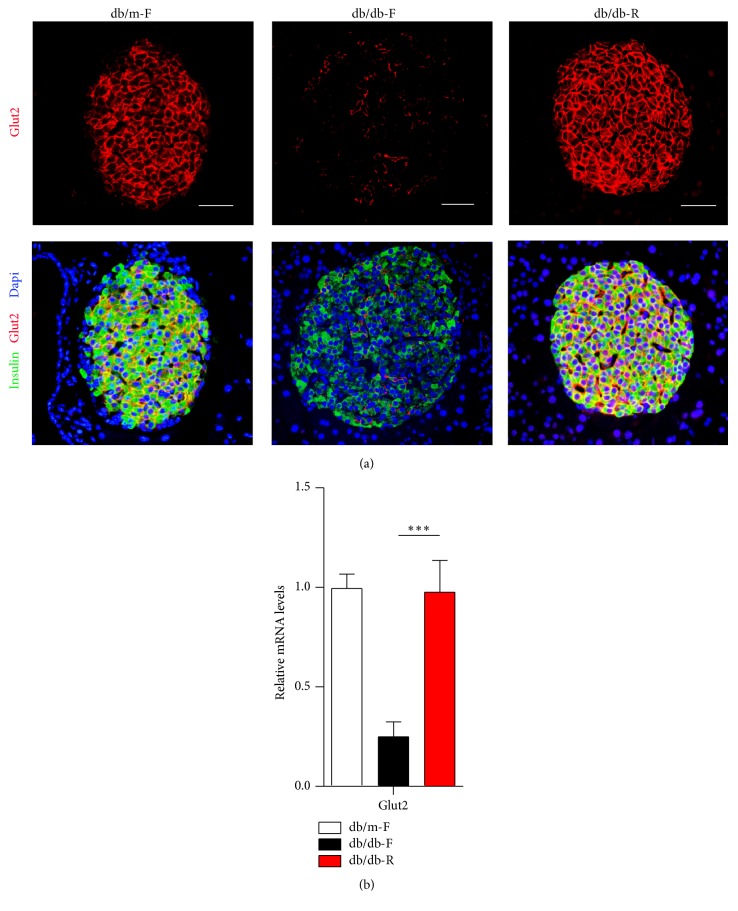
(a) Immunofluorescence analysis of Glut2 and insulin expression in islet cells in db/m-F, db/db-F, and db/db-R mice. (b) Real-time PCR analysis of Glut2 expression in the islets in db/m-F, db/db-F, and db/db-R mice. ^*∗∗∗*^ *P* < 0.001. The data shown represent three independent experiments. db/db-F and db/db mice given free access to regular chow; db/db-R and db/db mice receiving restricted food supply; db/m-F and db/m mice given free access to regular chow. Scale bars: 25 *μ*m.

Glut2 is the *β*-cell's principal glucose transporter and is essential for maintaining its function in insulin secretion [11]. Immunofluorescence staining showed that a near-complete loss in Glut2 expression was observed in db/db mice at 12 weeks of age before CR (data not shown). Intriguingly, Glut2 expression was significantly increased in db/db-R mice compared with db/db-F mice and returned to normal levels as in db/m-F mice (Figure 4(a)). Glut2 mRNA levels were also significantly increased in the islets of db/db-R mice compared with db/db-F mice (Figure 4(b)).

**Figure 5:**
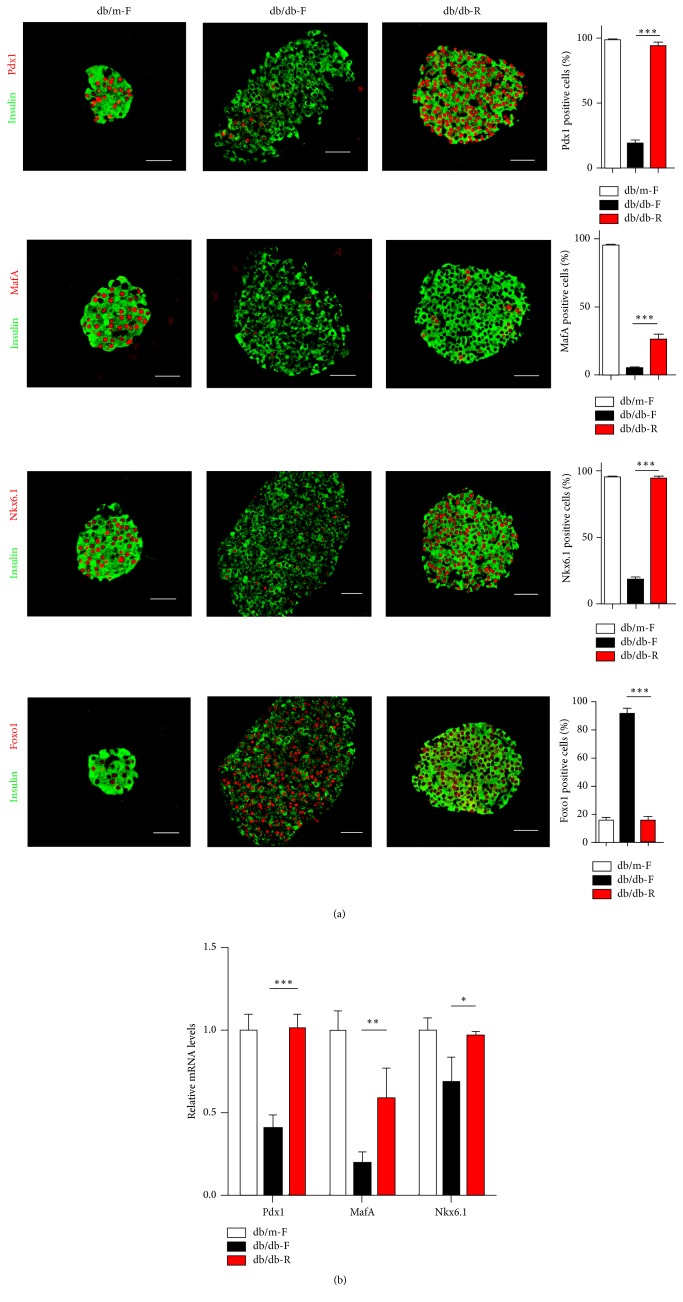
The expression levels of specific *β*-cell transcription factors in *β*-cells from db/m-F, db/db-F, and db/db-R mice. (a) Immunofluorescence analysis of Nkx6.1, Pdx1, MafA, and Foxo1 expression levels in islets from db/m-F, db/db-F, and db/db-R mice. The quantification of the percentage of *β*-cells containing these transcription factors is shown. (b) Real-time PCR analysis of Nkx6.1, Pdx1, and MafA expression in islets from db/m-F, db/db-F, and db/db-R mice. ^*∗*^ *P* < 0.05, ^*∗∗*^ *P* < 0.01, and ^*∗∗∗*^ *P* < 0.001. The data shown represent three independent experiments. db/db-F and db/db mice given free access to regular chow; db/db-R and db/db mice receiving restricted food supply; db/m-F and db/m mice given free access to regular chow. Scale bars: 25 *μ*m.

Nkx6.1 and Pdx1 have been shown to play important roles in *β*-cell differentiation, maturation, and function maintenance. Immunofluorescence staining showed that inactivation of Nkx6.1 and Pdx1 was observed in the islets of db/db-F mice, while their expression was significantly increased in db/db-R mice and returned to nearly normal levels in db/m-F mice (Figure 5(a)). MafA is a transcription factor that is tightly restricted to the *β*-cell nucleus in adult islets and is necessary for optimal insulin gene expression [12]. MafA expression was significantly increased in the islets of db/db-R mice compared with db/db-F mice (Figure 5(a)).

Transcription factor Foxo1 integrates signals enforcing *β*-cell fate under metabolic stress [13]. Immunostaining results showed that Foxo1 nuclear translocation was significantly increased in the islets of db/db-F mice and significantly reduced in db/db-R mice, which is similar to what was observed in db/m-F mice (Figure 5(a)). Moreover, the mRNA levels of* Nkx6.1*,* Pdx1*, and* MafA* were significantly higher in the islets of db/db-R mice compared with db/db-F mice (Figure 5(b)). The significant inactivation of Nkx6.1, Pdx1, and MafA expression observed in the islets of db/db mice at 12 weeks of age before CR (data not shown) suggested that the expression of these *β*-cell-specific TFs could be reversed by long-term CR.

### 3.5. The Expression of GLP-1-Pathway Associated Proteins

**Figure 6:**
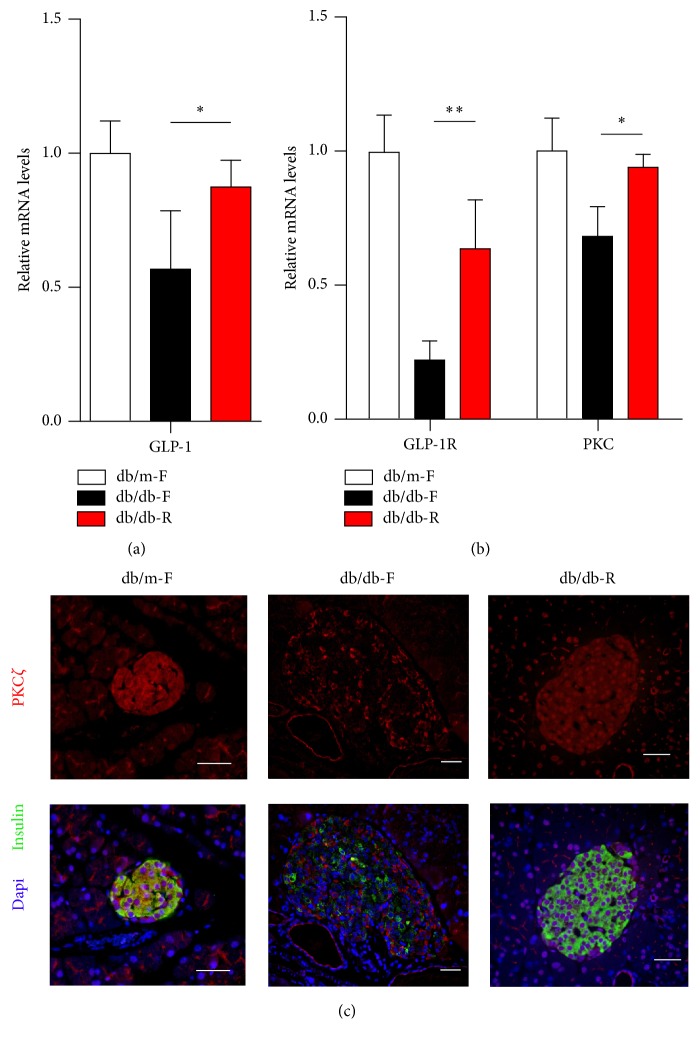
Real-time PCR analysis of* GLP-1* in the colonic tissue (a) and* GLP-1R* and* PKC* (b) in the islets in the db/m-F, db/db-F, and db/db-R mice. (c) Immunofluorescence analysis of PKC*ζ* expression levels in islets from db/m-F, db/db-F, and db/db-R mice.^*∗*^ *P* < 0.05, ^*∗∗*^ *P* < 0.01. The data shown represent three independent experiments. db/db-F and db/db mice given free access to regular chow; db/db-R and db/db mice receiving restricted food supply; db/m-F and db/m mice given free access to regular chow. Scale bars: 25 *μ*m.

The expression of* GLP-1* in colonic tissue was significantly reduced in the db/db-F mice, while its expression was significantly increased in the db/db-R mice (Figure 6(a)). Moreover, the mRNA levels of GLP-1 receptor (*GLP-1R*) and* PKC* in the islets were significantly increased in the db/db-R mice compared with the db/db-F mice (Figure 6(b)). Immunostaining results showed that PKC*ζ* was significantly reduced in the islets of db/db-F mice, while its total expression as well as nuclear translocation was significantly increased in the db/db-R mice (Figure 6(c)).

## 4. Discussion

It has previously been demonstrated that chronic hyperglycemia contributes to *β*-cell dedifferentiation and dietary restriction can preserve the function of pancreatic *β*-cells via cell kinetic regulation and suppression of oxidative/ER stress in db/db mice [14, 15]. Similarly, our results showed that *β*-cell dedifferentiation could be prevented or possibly reversed by long-term CR intervention, which might be regulated by a hierarchical network of TFs. Glut2 and Pdx1 are known as functional markers of mature *β*-cells [11, 16]. Loss of Glut2 cytoplasmic expression as well as Pdx1 nuclear expression is an early event associated with early-onset islet dysfunction [17]. In fact, Glut2 expression in islets is known to be regulated by Pdx1, and therefore the impaired expression of these two factors might have a common mechanism [18]. Nkx6.1 plays a critical role in the control of insulin biosynthesis, insulin secretion, and *β*-cell proliferation [19]. However, our results showed that Nkx6.1 expression is relatively intractable to change, as 62.6% of gene expression could be detected in db/db-F mouse islets. MafA is a master glucose-regulated TF that contributes to the maintenance of *β*-cell differentiation and controls, either directly or indirectly, the expression of target genes including Glut2 and Pdx1 [20, 21]. Intriguingly, a nearly complete loss in MafA expression was observed in db/db-F mouse islets and its levels were relatively low in db/db-R mice, suggesting that it is a “fragile” TF that is easy to compromise and intractable to restore. Transcription factor Foxo1 integrates signals regulating stress response [22]. During CR, translocation of Foxo1 to the nucleus was reduced, suggesting a reduction in oxidative stress. Foxo1 can protect against pancreatic *β*-cell failure through regulating MafA expression [23]. Loss of Foxo1 expression led to *β*-cell dedifferentiation [13]. However, there was no change in Foxo1 mRNA expression levels with and without CR. Similar results have been previously reported [6]. Thus, further studies are necessary to pinpoint the stage at which Foxo1 plays influential roles in *β*-cell dedifferentiation.

After the long-term CR, activation of the GLP-1 pathway was observed in our study, including increased expression of GLP-1 in the colonic tissue and GLP-1R and PKC in the islets. GLP-1 binds to GLP-1R and then regulates Pdx1 expression by PKC [24]. However, the upregulation of GLP-1 and GLP-1R could be the result of chronic euglycemia [25]. A study of double mutant LPR^−/−^; GLP-1R^−/−^ mice model may unveil the roles that GLP-1R signaling plays in *β*-cell function improvement after long-term CR.

## 5. Conclusion

In summary, an important feature of our findings was how characteristically Glut2, MafA, Pdx1, and Nkx6.1 were deactivated during *β*-cell dedifferentiation and how their expression was fully or partly reversed after CR intervention. Consequently, the identification of small molecules that increase the expression of these factors could be very useful in T2D treatment.
